# A Novel Tracking Algorithm via Feature Points Matching

**DOI:** 10.1371/journal.pone.0116315

**Published:** 2015-01-24

**Authors:** Nan Luo, Quansen Sun, Qiang Chen, Zexuan Ji, Deshen Xia

**Affiliations:** School of Computer Science and Engineering, Nanjing University of Science and Technology, Nanjing, Jiangsu, China; Nanjing University of Aeronautic and Astronautics, CHINA

## Abstract

Visual target tracking is a primary task in many computer vision applications and has been widely studied in recent years. Among all the tracking methods, the mean shift algorithm has attracted extraordinary interest and been well developed in the past decade due to its excellent performance. However, it is still challenging for the color histogram based algorithms to deal with the complex target tracking. Therefore, the algorithms based on other distinguishing features are highly required. In this paper, we propose a novel target tracking algorithm based on mean shift theory, in which a new type of image feature is introduced and utilized to find the corresponding region between the neighbor frames. The target histogram is created by clustering the features obtained in the extraction strategy. Then, the mean shift process is adopted to calculate the target location iteratively. Experimental results demonstrate that the proposed algorithm can deal with the challenging tracking situations such as: partial occlusion, illumination change, scale variations, object rotation and complex background clutter. Meanwhile, it outperforms several state-of-the-art methods.

## Introduction

The target tracking technology is an important issue in computer vision tasks especially for the applications such as intelligent video surveillance [1, 2], automatic driving [3], vision-based control [4], the video-based reconstruction [5], video-based human interaction [6], and etc. The aim of target tracking is to find out a continuous correspondence of the target region in each frame.

As a nonparametric density estimator, the mean shift algorithm was introduced in 1975 [7], which was used to compute the nearest mode of a sample distribution iteratively [8]. Due to its simplicity in implementation and robustness to occlusion and distortion, mean shift becomes the most popular target tracking method. In the classical kernel based mean shift tracking algorithm (KMS [9, 10]), color histogram is used to describe the target, and Bhattacharyya coefficient is employed to measure the similarity between the target model and the target candidates. Location of the target is achieved by finding the local minimum of the distance measure function iteratively. Based on the mean shift framework, many algorithms combining with feature extensions or adaptive scale have been proposed. In [11], the target size was determined by executing the algorithm three times in different scales. Then, an expectation maximization based mean shift tracking algorithm (EMshift) was proposed in [12], which simultaneously estimated the position of the local mode and the covariance matrix. Besides, in [13], a new histogram called Spatiogram that included potentially higher order moments was used in the mean shift frame to increase robustness in tracking. Meanwhile, a weighted fragment based approach that tackled partial occlusion was proposed in [14], where the weights were derived from the difference between the fragment and background colors. According to the hypothesis testing, Peng et al. [15] proposed a mean shift tracking algorithm using Kalman filter to update histogram bins. It was widely considered that the cross-bin metrics which employed a bin-by-bin metric [16] were generally more robust for measuring histograms similarity than the traditional mean shift algorithm. Therefore, these works using the earth mover’s distance (EMD) as similarity measurement showed their advantage in [16–18]. An adaptive mean shift tracking (SOAMST) algorithm was proposed in [19] to address the problem of how to estimate the scale and orientation changes of the target in the mean shift. In [20], Ning et al. demonstrated that the background-weighted histogram (BWH) algorithm proposed in [10] did not introduce any additional information to improve the tracking effect. In addition, a corrected BWH (CBWH) formula by transforming the target model was proposed which could effectively reduce the interference of background in target localization.

However, the color histogram based algorithms are sensitive to similar backgrounds and illumination variation. Therefore, some robust and distinguishing features such as SIFT [21] and SURF [22] are introduced into the target tracking field. Zhou et al. [23] proposed an algorithm which used the EM algorithm to search for an appropriate similarity region. Meanwhile, the distance between the detected locations by mean shift and SIFT correspondence is minimized respectively. Haner et al. [24] proposed a method to improve the robustness of mean shift tracking algorithm by using SURF features. Zhang et al. [25] utilized SIFT feature points which were extracted from the search region to construct the color histogram and color probability distribution, then the target location was guided by matching these two sets of features. All above-mentioned methods demonstrate that the distinguishing features can be used to make the color histogram based algorithms more robust. However, most of those methods are time consuming due to their features detection and description strategies. With the development of image local features, more and more new detectors and descriptors have been proposed in recent years, such as FAST [26], AGAST [27], BRIEF [28], BRISK [29], ORB [30], FREAK [31] and DAISY [32]. These methods are considered to be more efficient in terms of speed and matching performance. Besides, in [33], feature histograms were extracted from a sparse set of SIFT feature points by using k-means clustering. Significant performance could be achieved by training these histograms in a SVM classifier for texture classification and object categories. Hence we are inspired to use the latest and faster features to create histograms in the mean shift iteration framework to locate the target.

In this paper, an effective target tracking algorithm is proposed to cope with the complex tracking. Comparing with other blobs detector such as SIFT and SURF, FAST [26] is a corner based detector with high speed which can get more feature points. Therefore, the statistical histogram is more reasonable and preciser with more feature points. As mentioned above, binary features such as BRIEF [28], BRISK [29], ORB [30] and FREAK [31] have fast speed due to the binary stream. However, they are proposed for embedded devices with small memory and low computation complexity like smart phone or tablet PC. Hence the matching ability of these descriptors is not as good as the traditional ones [34]. In addition, the DAISY descriptor is chosen to describe the feature which has been demonstrated with better performance in numerous image distortions [35]. Moreover, due to its simple architecture, the DAISY descriptor which can be efficiently applied to the framework of 3D reconstruction. Therefore, the proposed algorithm uses FAST [26] to detect the feature points and utilizes the DAISY descriptor [32, 36] to describe the points. Then we create the histograms both of the target model and the candidate using the feature points and find the correspondence between two frames. Finally, we calculate the new location of the target by matching the histograms in the mean shift framework.

It should be noted that this paper is an extension of our previous work [37]. Specifically, the extensions include:

1) Feature points matching strategy is introduced to the mean shift tracking process.
2) A novel target representation histogram is proposed by using k-means cluster.
3) Much more tracking results are shown on more public datasets.

The remainder of this paper is organized as follows. In the section Method, a mean shift based algorithm using FAST detector and DAISY descriptor for object tracking is proposed. Section Experiments shows the comparison results of several state-of-the-art methods and the proposed algorithm. Section Conclusions concludes this paper in the end.

## Methods

### Mean shift algorithm based on kernel theory

In the mean shift algorithm, the color histogram of the target region is adopted as the template description. Let {*x*_*i*_}, *i* = 1, ⋯, *n* be the pixel locations of the region defined as the target model. The coordinates of the target center is *x*_0_, and the set is normalized according to the bounding box bandwidth *h*. Then the color distribution is discretized into *m*− bins histogram. The function *b*: *R* ^2^ → {1, ⋯, *m*} associates the pixel at location *x*_*i*_ to the index *b*(*x*_*i*_) of its bin in the feature space.

To all the pixels in the target region of the first frame, computing the histogram is called the target model description. Moreover, *x*_*i*_ is the *i*−th pixel in the target region, and the *m*−bin color histogram is *q*_*u*_ = {*q̂*_*u*_(*y*)}, *u* = 1, ⋯, *m*, which can be stated as:

$${\hat{q}}_{u}=C\sum_{i=1}^{n}k\left({\left\|\frac{x_{i}-x_{0}}{h}\right\|}^{2}\right)\delta \left[b\left(x_{i}\right)-u\right]$$ (1)

where *n* is the pixel number of the target model area, *h* is the window bandwidth, and *δ* is the Kronecker delta function. As a convex monotonically decreasing function, kernel function *k*(*x*) assigns smaller weights to pixels farther from the target center. *C* is the normalization constant to insure the condition $\sum_{u=1}^{m}{\hat{q}}_{u}=1$, which is derived from:

$$C=\frac{1}{\sum_{i=1}^{n}k\left({\left\|\frac{x_{i}-x_{0}}{h}\right\|}^{2}\right)}$$ (2)

Similarly, let $\{x_{i}^{*}\},i=1,\cdots ,n$ be the pixel locations of the target candidate which is centered at *y* in the current frame. The color histogram of the target candidate template is *p*_*u*_ = {*p̂*_*u*_(*y*)}, *u* = 1, ⋯, *m*, which can be described as:

$${\hat{p}}_{u}\left(y\right)=C_{k}\sum_{i=1}^{n_{h}}k\left({\left\|\frac{x_{i}^{*}-y}{h}\right\|}^{2}\right)\delta \left[b\left(x_{i}^{*}\right)-u\right]$$ (3)

where

$$C_{k}=\frac{1}{\sum_{i=1}^{n}k\left({\left\|\frac{y-x_{i}^{*}}{h}\right\|}^{2}\right)}$$ (4)

is also the normalization constant. It is obvious that that *C*_*k*_ does not rely on *y*, because the pixel locations $x_{i}^{*}$ are organized in a regular lattice and *y* is one of the lattice nodes [10]. Therefore, *C*_*k*_ can be calculated in advance when the kernel density and the value of bandwidth *h* are given.

The Bhattacharyya coefficient [38] is used to measure the similarity between the histograms which is described as:

$$\hat{\rho }\left(y\right)\equiv \rho \left[\;\hat{p}\left(y\right),\hat{q}\right]=\sum_{u=1}^{m}\sqrt{\;\hat{p}\left(y\right)\hat{q}}$$ (5)

To find the position corresponding to the target in the current frame, the Bhattacharyya coefficient Eq. 5 should be maximized as a function of *y*. More involved details can be found in [39]. The brief derivation process is shown as follows:

Let the target location in the previous frame *ŷ*_0_ be the initial position of the target candidate in the current frame. Use the Taylor expansion around the *p̂*_*u*_(*ŷ*_0_), the linear approximation of Bhattacharyya coefficient is obtained as

$$\rho \left[\;\hat{p}\left(y\right),\hat{q}\right]\approx \frac{1}{2}\sum_{u=1}^{m}\sqrt{\;{\hat{p}}_{u}\left({\hat{y}}_{0}\right){\hat{q}}_{u}}+\frac{1}{2}\sum_{u=1}^{m}{\hat{p}}_{u}\left(y\right)\sqrt{\;\frac{{\hat{q}}_{u}}{{\hat{p}}_{u}\left({\hat{y}}_{0}\right)}}$$ (6)

Recalling Eq. 3 yields

$$\rho \left[\;\hat{p}\left(y\right),\hat{q}\right]\approx \frac{1}{2}\sum_{u=1}^{m}\sqrt{\;{\hat{p}}_{u}\left({\hat{y}}_{0}\right){\hat{q}}_{u}}+\frac{C_{k}}{2}\sum_{i=1}^{n_{h}}\omega_{i}k\left({\left\|\frac{x_{i}-y}{h}\right\|}^{2}\right)$$ (7)

where

$$\omega_{i}=\sum_{u=1}^{m}{\hat{p}}_{u}\left(y\right)\sqrt{\;\frac{{\hat{q}}_{u}}{{\hat{p}}_{u}\left({\hat{y}}_{0}\right)}}\delta \left[b\left(x_{i}\right)-u\right]$$ (8)

In order to maximize Eq. 5, the second term of equation 7 must be maximized, since the first term is independent of *y*. Notice that the second term represents the density estimation with kernel function *k*(*x*) at *y* in the current frame. The maximum of this density mode in the local neighborhood can be found by using the mean shift process [10]. In this procedure, the kernel is recursively moved from the current location *ŷ*_0_ to the new location *ŷ*_1_ using the following function:

$${\hat{y}}_{1}=\frac{\sum_{i=1}^{n_{h}}x_{i}\omega_{i}g\left({\left\|\frac{x_{i}-{\hat{y}}_{0}}{h}\right\|}^{2}\right)}{\sum_{i=1}^{n_{h}}\omega_{i}g\left({\left\|\frac{x_{i}-{\hat{y}}_{0}}{h}\right\|}^{2}\right)}$$ (9)

where *g*(*x*) = −*k* ^′^(*x*) and the new location *ŷ*_1_ is the center of the target region in the current frame.

### Feature extraction strategy

FAST detector is proposed by Rostenand and Drummond in 2006 [26], and they are widely used because of their computational properties. FAST features are described as a sufficient number of pixels which have different gray value scales around the center point. In this paper, we adopt FAST-9 (circular radius of 9), which has good performance in [30].

DAISY descriptor proposed by Tola [32, 36] is a new image local feature descriptor in recent years. The core concept of DAISY is using multiple scale Gaussian filter functions to convolve with several gradient maps of the original image. Since Gaussian filter functions have separability, this descriptor shows its efficiency and effectiveness in dense matching task.

First, for input image *I*, *H* number of orientation maps are computed with pixel difference, and they are denoted by

$$G_{o}={\left(\frac{\partial I}{\partial o}\right)}^{+},1\leq o\leq H$$ (10)

where (.)^+^ means the operator such that (*a*)^+^ = *max*(*a*, 0).

Then, the orientation maps are convolved with several Gaussian filter functions under different Σ values to obtain the convolved orientation maps for different regions. These convolved orientation maps are denoted by

$$G_{o}^{\Sigma }=G_{\Sigma }*{\left(\frac{\partial I}{\partial o}\right)}^{+}$$ (11)

**Figure 1:**
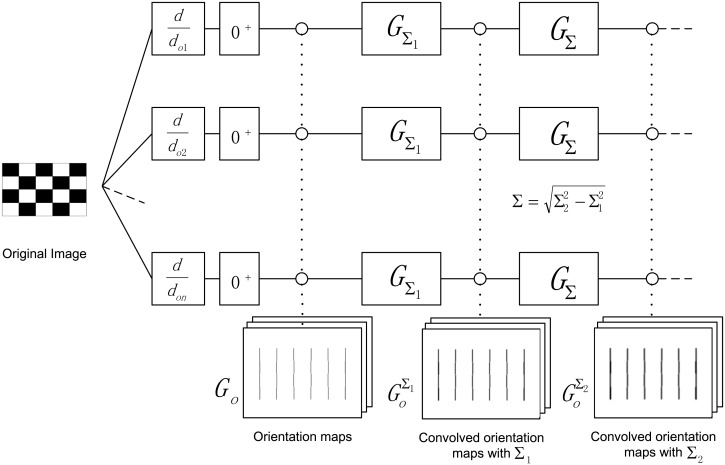
The calculation process of the convolved orientation maps in DAISY.

In addition, the convolved orientation maps can be computed in an efficient way to reduce the computational complexity since the Gaussian filters are separable. That is, a lager Gaussian filter Σ_2_ is obtained from consecutive convolution with the smaller one Σ_1_ using the following equation:

$$G_{o}^{\Sigma_{2}}=G_{\Sigma_{2}}*{\left(\frac{\partial I}{\partial o}\right)}^{+}=G_{\Sigma }*G_{\Sigma_{1}}*{\left(\frac{\partial I}{\partial o}\right)}^{+}=G_{\Sigma }*G_{o}^{\Sigma_{1}}$$ (12)

where $\Sigma =\sqrt{\Sigma_{2}^{2}-\Sigma_{1}^{2}}$. The whole calculation process of the convolved orientation maps is shown intuitively in Fig. 1.

**Figure 2:**
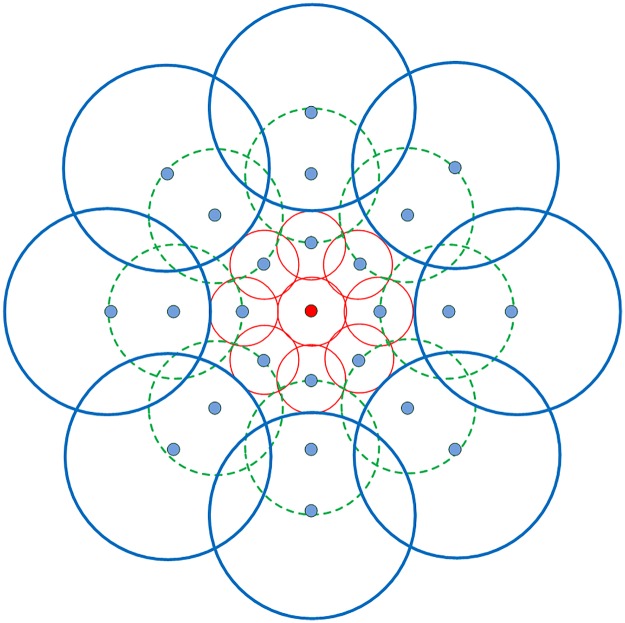
DAISY descriptor structure.

As shown in Fig. 2, DAISY descriptor has a central-symmetrically architecture which is similar to the flower daisy [32, 36]. This is the origin of its name. The parameters settings and the proposed values are listed in Table 1. DAISY uses the circular grids rather than the rectangular ones which are utilized in SIFT and SURF, and it demonstrates that this kind of structures has better localization properties than the rectangular ones [40]. In DAISY structure, the red point denotes the location of the center pixel, and the blue ones represent the sample points in outer rings. Each circle contains one histogram vector which is made of the convolved orientation maps in different gradient directions of this region, where the amount of Gaussian smoothing is proportional to the radii of the circle. Therefore, for a certain pixel at location (*u*, *v*) in the given image, let $G_{o}^{\Sigma_{i}}(u,v)$ represent the gradient norm which has been convolved with Gaussian filter Σ_*i*_ for direction *o*. The histogram of the location can be represented by an *H*− dimensional vector as

$$h_{\Sigma }\left(u,v\right)={\left[G_{1}^{\Sigma }\left(u,v\right),\cdots ,G_{H}^{\Sigma }\left(u,v\right)\right]}^{T}$$ (13)

Then each vector is normalized to unit form, and denoted by ${\tilde{h}}_{\Sigma }(u,v)$. The normalization is performed independently in each histogram in order to avoid the errors caused by occlusions and different viewpoints. Finally, the DAISY descriptor of a certain pixel (*u*, *v*) can be formed by connecting the previously computed normalized vectors of the center points and its neighbor sample points of outer rings:

$$D\left(u,v\right)=\left[\begin{array}{c} {\tilde{h}}_{\Sigma_{1}}^{T}\left(u,v\right)\\ {\tilde{h}}_{\Sigma_{1}}^{T}\left(l_{1}\left(u,v,R_{1}\right)\right),\cdots ,{\tilde{h}}_{\Sigma_{1}}^{T}\left(l_{T}\left(u,v,R_{1}\right)\right)\\ {\tilde{h}}_{\Sigma_{2}}^{T}\left(l_{1}\left(u,v,R_{2}\right)\right),\cdots ,{\tilde{h}}_{\Sigma_{2}}^{T}\left(l_{T}\left(u,v,R_{2}\right)\right)\\ \cdots \\ {\tilde{h}}_{\Sigma_{Q}}^{T}\left(l_{1}\left(u,v,R_{Q}\right)\right),\cdots ,{\tilde{h}}_{\Sigma_{Q}}^{T}\left(l_{T}\left(u,v,R_{Q}\right)\right) \end{array}\right]$$ (14)

where, *l*_*j*_(*u*, *v*, *R*) is the location with distance *R* from (*u*, *v*) in the *j*−th direction. In this paper, considering the computational efficiency, our DAISY implementation uses the following parameters: *R* = 10, *Q* = 2, *T* = 8, *H* = 8, which means the dimensionality of the feature vector is *D* = (*Q*×*T* + 1)×*H* = 136.

**Table 1:** DAISY Parameters.

Symbol	Description and Default Value
*R*	Distance from the center pixel to the outer most grid point. (15)
*Q*	Number of convolved orientations layers with different Σ^′^ *s*. (3)
*T*	Number of histograms at a single layer. (8)
*H*	Number of bins in the histogram. (8)
*S*	Number of histograms used in the descriptor = *Q* × *T* + 1.
*D*_*S*_	The total size of the descriptor vector = *S* × *H*.

### Proposed method

**Definition:** All the variables used in this paper are defined as follows: *I*_*t*_ denotes the *t*−th frame of the video sequence, where *t* = 0,1, ⋯, *N*. The target region is selected manually with a rectangle on the first frame *I*_0_ which is defined as *R*_0_, in which the rectangle is represented with a center *c*_0_ = (*u*_0_, *v*_0_), a width *w*_0_ and a height *h*_0_. Then we can compute the FAST feature points and the DAISY descriptors which are denoted as set $S_{t}=\{(x_{t}^{1},d_{t}^{1}),(x_{t}^{2},d_{t}^{2}),\cdots ,(x_{t}^{n},d_{t}^{n})\}$, where *n* is the number of feature points, $x_{t}^{j}=(u_{t}^{j},v_{t}^{j})$ marks location of the *j*−th point and its DAISY descriptor $d_{t}^{j}$ in frame *I*_*t*_. Therefore, the whole tracking problem can be defined as: given *I*_*t*_, *R*_*t*_, *S*_*t*_ and *I*_*t*+1_, the task is to find out the *R*_*t*+1_. That means to determine a new center *c*_*t*+1_ = (*u*_*t*+1_, *v*_*t*+1_) and new edges *w*_*t*+1_, *h*_*t*+1_ of *R*_*t*+1_.

**Figure 3:**
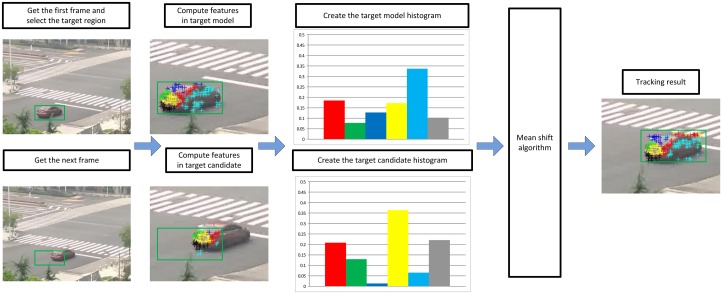
Flow diagram of the proposed algorithm in an example.

In the first frame, we detect the FAST feature points in the target region and use them to form the target model histogram. Then k-means clustering algorithm is applied to divide these feature points into several bins according to their DAISY descriptors. Next, in the current frame, the FAST feature points are detected in the target candidate region. After that, we utilize these points to create the target candidate histogram and then find the corresponding points between two frames. At last, these two histograms and the corresponding points are imported into the mean shift framework to compute the location of the target. Fig. 3 shows a flow diagram of the proposed algorithm as an example. In the next part of this section the proposed method is described in detail.

**Target model histogram:** First of all, the feature points in the target region *R*_0_ are obtained by the FAST detector and the DAISY descriptor. Then all the DAISY descriptors are clustered into *M* bins by using the conventional k-means algorithm, where the *M* cluster centers are *C*_*u*_, *u* = 1, 2, ⋯, *M*,. Let *c*_0_ be the center of the target region, and *S*_0_ be the set of feature points. Since each feature point $x_{0}^{j},j=1,2,\cdots ,m_{0}$ can only belong to one cluster, we can describe the target model $q_{u}=\{\tilde{q_{u}}(y)\},u=1,2,\cdots ,M$ as follow:

$$\tilde{q_{u}}=C\sum_{j=1}^{m_{0}}k\left({\left\|\frac{x_{0}^{j}-c_{0}}{h}\right\|}^{2}\right)\delta \left[Idx\left(x_{0}^{j}\right)-u\right]$$ (15)

where *m*_0_ is the total number of the feature points in the target region, *h* is the bandwidth, *δ* is the Kronecker delta function and $Idx(x_{0}^{j})$ returns the cluster index of the feature point $\left(x_{0}^{j}\right)$. In our method, we use 2*D* Gaussian function to implement the kernel function *k*. Similar with Equation 1, *C* is the normalization constant to make sure that $\sum_{u=1}^{M}\tilde{q_{u}}=1$.

**Figure 4:**
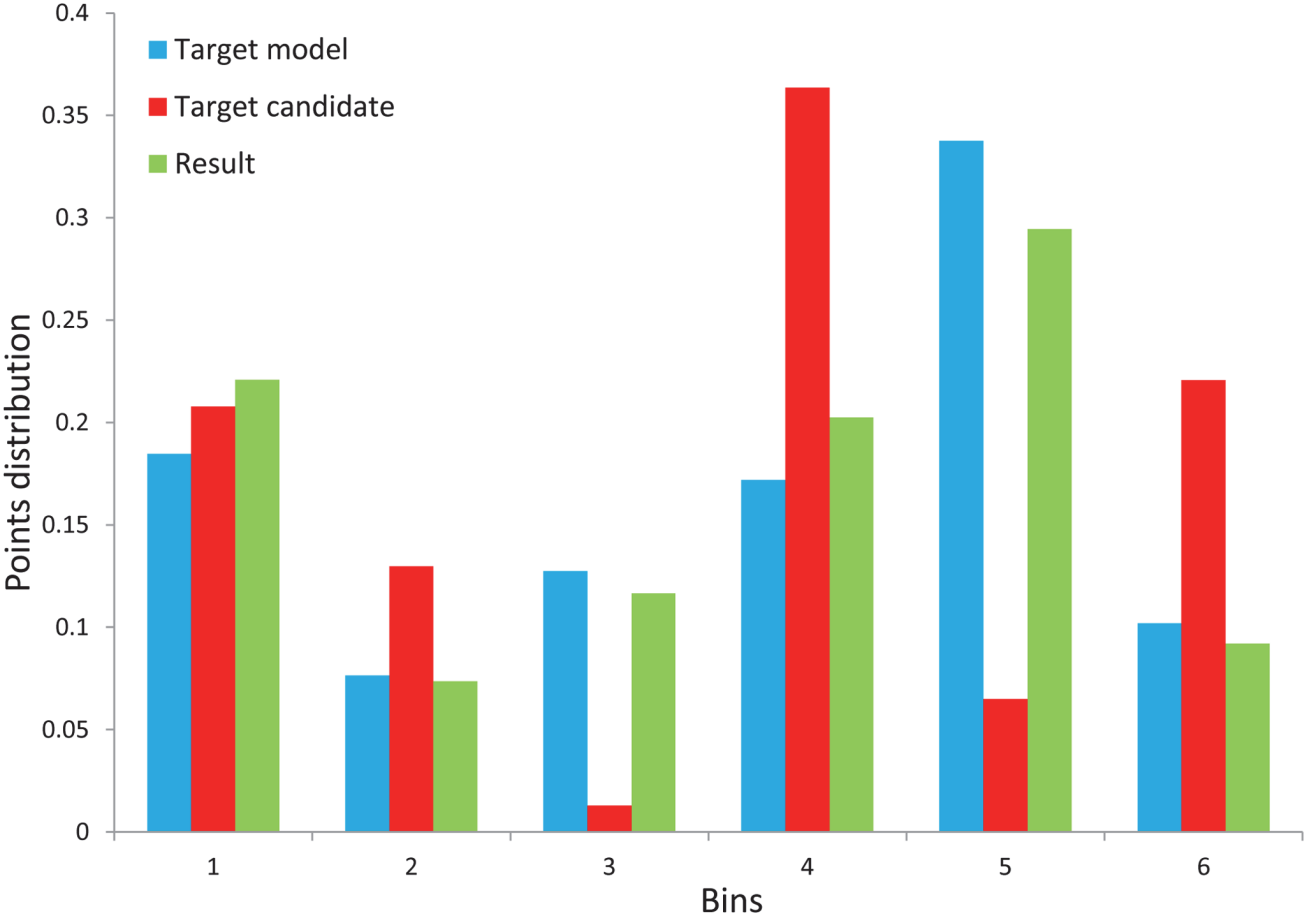
Target Histograms of the example in Fig. 3.

**Target candidate histogram:** Since the target model $\tilde{q_{u}}$ is computed, we can get the target candidate histogram in the same way. Firstly, in current frame *I*_*t*_, the feature points *S*_*t*_ are computed in the result region *R*_*t*−1_ from previous frame. Here, we do not need to run the k-means algorithm again to get the cluster results. Each feature point belongs to a particular cluster center *C*_*u*_, *u* = 1, 2, ⋯, *M* which is computed in the first frame when its Euclidean distance to this center is minimum. And then the distribution of the target candidate $p_{u}=\{\tilde{p_{u}}(y)\},u=1,2,\cdots ,M$ can be written as:

$$\tilde{p_{u}}=C_{h}\sum_{j=1}^{m_{t}}k\left({\left\|\frac{x_{t}^{j}-y}{h}\right\|}^{2}\right)\delta \left[Idx\left(x_{t}^{j}\right)-u\right]$$ (16)

where *m*_*t*_ is the length of *S*_*t*_ and *C*_*h*_ is the normalization constant. Since the mean shift algorithm is based on histogram matching, more distinguishing histograms leads to better results. Fig. 4 shows the histograms difference of the example in Fig. 3, where the histograms contain 6 bins representing 6 clusters. The histogram in blue represents the target model region of the example in Fig. 3. The red one is the histogram of the target candidate region at the beginning of mean shift process. The last one in green is the histogram of the result when mean shift iteration process ends. From Fig. 4 we can obviously find that the histograms of the target model and the tracking result are analogous. Meanwhile, the histogram from target candidate region and the other two are significantly different. It is proved that the presentation of the target in the proposed method has strong distinctiveness.

**Matching strategy:** In this paper, the nearest neighbor ratio matching strategy [41] is adopted for the features matching. For each featrue point in the target model region, its DAISY descriptor has been matched with each featrue point in the target candidate region and the Euclidean distances are computed. When the ratio of the minimum distance and the second minimum one is less than a threshold value, which is usually set as 0.7, we consider the point with the minimum distance is the corresponding point.

**Location:** The location of the tracking target can be obtained by the following function:

$$c_{t}=\frac{\sum_{j=1}^{m}x_{t}^{j}\omega_{j}g\left({\left\|\frac{x_{t}^{j}-c_{t-1}}{h}\right\|}^{2}\right)}{\sum_{j=1}^{m}\omega_{j}g\left({\left\|\frac{x_{t}^{j}-c_{t-1}}{h}\right\|}^{2}\right)}$$ (17)

where *m* is the number of corresponding points and *g*(*x*) = −*k* ^′^(*x*) is the derivative of the kernel function. $\omega_{j}=\sum_{u=1}^{m}{\hat{p}}_{u}(y)\sqrt{\;\frac{{\hat{q}}_{u}}{{\hat{p}}_{u}({\hat{y}}_{0})}}\delta [Idx(x_{t}^{j})-u]$ is the weight with each feature point.

**Scale strategy:** A simple scale factor is computed by using pair-wise corresponding points in the proposed method. Assuming that the scale changes between frames in anisotropic manner, which means there are two scaling factors *s*_*x*_, *s*_*y*_ presenting the *x*, *y* coordinate respectively. Then they can be defined as:

$$s_{x}=C_{x}\sum_{i=1}^{k}\sum_{j=i+1}^{k}\frac{x_{t}^{i}.x-x_{t}^{j}.x}{x_{t-1}^{i}.x-x_{t-1}^{j}.x}$$ (18)

where *k* is the number of corresponding points, $x_{t}^{i}$ and $x_{t-1}^{i}$ are one pair of corresponding points in two neighbor frames. Moreover, $x_{t}^{i}.x$ means the coordinate value of point $x_{t}^{i}$ in x-axis, the combination between *i* and *j* is $C_{k}^{2}$, therefore we set $C_{x}=1/C_{k}^{2}$ to compute the average value of all of the scale factors. Meanwhile, *s*_*y*_ can be computed using the same way.

**Searching extending strategy:** There are two reasons for the target being missing in the tracking process. One is that the target moves too fast to stay in the target candidate region in the current frame, the other is the partial or full occlusions. In our experiments, these situations will result in that there is no detecting feature point or no corresponding point. In order to solve this problem, the searching extending strategy is proposed in this paper. When the no point situation occurs, the bandwidth of the original target candidate region becomes twice. Then the feature points detected in the new candidate region are utilized to calculate the new candidate histogram. It is an effective way to capture the fast moving target or to deal with the partial occlusion. Moreover, if full occlusion occurs, the bandwidth of the region will keep the same instead of changing larger to introduce errors.

Thus, the proposed mean shift based algorithm using DAISY descriptor for target tracking can be listed as follows:

**Algorithm 1**: Mean shift based tracking algorithm using DAISY descriptor

**while** *Load video frame I*_*t*_, *t* = 0, 1, 2, ⋯, *N* **do**

**if** *t* = 0 **then**

   1. Select target region *R*_0_ manually

   2. Extract FAST feature points and DAISY descriptor in *R*_0_

   3. Cluster the descriptors into *M* bins using k-means algorithm

   4. Use Equation 15 to compute the target model histogram

**end**

**else**

   5. *step* = 0, *extendFlag* = 0

**while** *step* < *MaxStep* **do**

    6. *step* = *step* + 1

    7. Compute FAST feature points and DAISY descriptor in *R*_*t*−1_

    8. Use Equation 16 to compute the target candidate histogram

    9. Find out the corresponding points using the nearest neighbor ratio matching strategy

**if** *(No feature point **OR** No corresponding point) **AND** extendFlag* == *0* **then**

     10. extendFlag = 1

     11. Update *R*_*t*−1_ using searching extending strategy

     12. Goto 7

**end**

    13. Compute new target region center *c*_*t*_ using Equation 17

**if** ‖*c*_*t*_−*c*_*t*−1_‖ ≤ *ε* **then**

     14.break

**end**

    15. Update *R*_*t*−1_ according to *c*_*t*_

**end**

   16. Compute scale factor using Equation 18

   17. Update new target bandwidth

   18. Update new target region *R*_*t*_

**end**

**end**

## Experimental Results

In this paper, we collect 20 sequences containing different situations from published literature [37, 42–50]. All the datasets and the ground truth are available on these websites:

1. http://www.lanrongyi.com/data/dataset.zip [37];
2. http://www.visual-tracking.net/ [42].

These sequences contain the most challenging attributions in object tracking: partial occlusion(OCC), illumination variation(IV), scale variations(SV), in-plane and out-of-plane rotation(IPR and OPR), non-rigid object deformation(DEF), motion blur(MB), fast motion(FM), background clutters(BC) and low resolution(LR) [42]. More details about the data are shown in Table 2. In addition, the full occlusion is not considered in this paper because the mean shift algorithm cannot deal with this situation [10].

**Table 2:** The information of 20 sequences. (Attributions: OCC—partial occlusion; IV—illumination variation; SV—scale variations; IPR—in-plane rotation; OPR—out-of-plane rotation; DEF—non-rigid object deformation; MB—motion blur; FM—fast motion; BC—background clutters; LR—low resolution.)

Sequence	Frame numbers	Resolution	Attributions	Source
Basketball	725	576 × 432	IV, OCC, DEF, OPR, BC	[46]
Board	461	640 × 480	SV, OCC, FM, MB, BC	[47]
Boy	602	640 × 480	SV, MB, FM, IPR, OPR	[42]
Car	86	320 × 204	BC, LR	[37]
Caviar	382	384 × 288	SV, OCC, LR	[44]
Couple	140	320 × 240	SV, DEF, FM, OPR, BC	[48]
Crossing	120	360 × 240	SV, DEF, FM, OPR, BC	[42]
Deer	71	704 × 400	MB, FM, IPR, BC, LR	[46]
Doll	3872	400 × 300	IV, SV, OCC, IPR, OPR	[42]
Football	74	352 × 288	IPR, OPR, BC	[42]
Girl	500	128 × 96	SV, OCC, IPR, OPR	[50]
Jumping	313	352 × 288	MB, FM	[48]
MountainBike	228	640 × 360	IPR, OPR, BC	[49]
Occlusion	898	352 × 288	OCC	[42]
Singer	351	624 × 352	IV, SV, OCC, OPR	[46]
Subway	175	352 × 288	OCC, DEF, BC	[42]
Sylvester	1344	320 × 240	IV, IPR, OPR	[43]
Tiger	365	640 × 480	IV, OCC, DEF, MB, FM, IPR, OPR	[45]
Walking	412	768 × 576	SV, OCC, DEF	[42]
Walkman	104	276 × 206	SV, DEF, OPR	[37]

The proposed algorithm is compared with the state-of-the-art tracking algorithms based on mean shift, including KMS [10], EMshift [12], SOAMST [19] and CBWH [20]. Meanwhile, several non-mean shift tracking algorithms are also involved in our experiments, which are ASLA [51], SCM [52] and CT [53]. For a fair evaluation, parameters for the algorithms are left in default as set by the authors. In our implementation of the proposed algorithm, we chose FAST-9 (circular radius of 9) in feature points detection. Moreover, the number of clusters *M* is set to 6 in all the experiments in this paper. We also limit the number of mean shift iterations *MaxStep*, which is 15. It should be noted that the average number of iterations is about 3 in practice. The proposed algorithm is implemented on a 1.6Ghz Intel(R) Pentium(R) PC and the software environment is MATLAB.

### Quantitative Evaluation

**Figure 5:**
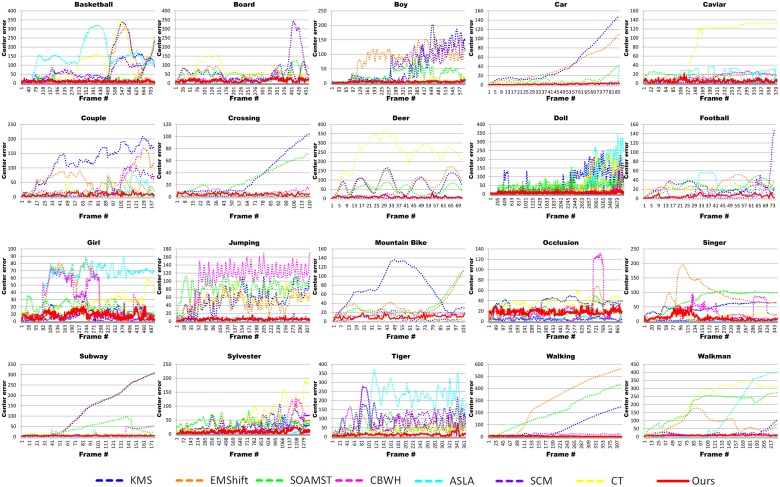
Quantitative evaluation in terms of center location error (in pixel).

The performance of the above-mentioned algorithms is evaluated by using two criteria: the center location error and the overlap rate, which are both computed against manually labeled ground truth. Table 3 lists the average center location errors in pixels of each sequence, where a smaller value means a more accurate result. In order to be more intuitive, the line charts of the center location error of each sequence are shown in Fig. 5. The evaluation of overlap rate is defined as:

$$Overlap=\frac{area(R_{T}\cap R_{G})}{area(R_{T}\cup R_{G})}$$ (19)

where the region *R*_*T*_ is the tracking result bounding box and the region *R*_*G*_ is the ground truth one in each frame. Table 4 reports the average overlap rates, where a larger value means a better result. Obviously, the proposed algorithm performs well against the mentioned state-of-the-art methods.

**Table 3:** Average center location error (in pixel). The best two results are shown in **Bold** and *Italics* fonts.

Algorithms	KMS [10]	EMshift [12]	SOAMST [19]	CBWH [20]	ASLA [51]	SCM [52]	CT [53]	Our
Basketball	80.74	55.58	25.43	*18.07*	150.51	62.51	102.87	**10.53**
Board	44.11	47	44.72	21.5	**13.05**	20.80	68.81	*14.57*
Boy	55.4	81.41	29.28	*3.88*	**3.63**	54.71	6.90	4.89
Car	46.04	38.54	9.78	*2.87*	3.04	4.82	5.42	**2.35**
Caviar	3.48	14.85	12.14	14.75	15.31	**0.87**	82.58	*2.72*
Couple	111.35	52.53	14.05	27.13	*9.31*	79.03	17.06	**8.25**
Crossing	34.05	4.16	30.25	5.79	*1.69*	**1.44**	2.48	4.63
Deer	74.83	83.46	45.18	10.76	*8.47*	10.8	17.74	**7.20**
Football	67.96	26.10	45.13	9.47	51.43	*8.57*	51.18	**8.51**
Doll	20.83	26.85	24.75	*7.84*	19.55	15.80	24.59	**5.63**
Girl	11.52	32.75	18.98	28.83	58.03	*9.80*	22.69	**8.50**
Jumping	54.26	46.24	85.10	105	*4.65*	**3.91**	52.82	5.40
MountainBike	22.05	77.37	190.8	10.60	126.48	*10.24*	219.43	**9.18**
Occlusion	32.13	20.67	20.47	22.53	*7.47*	**4.13**	35.69	12.24
Singer	49.37	87.11	75.4	31.63	**3.23**	*5.88*	15.76	12.30
Subway	129.65	131.12	39.54	13.59	*4.16*	**4.04**	11.65	5.43
Sylvester	24.84	30.01	37.99	22.45	*10.58*	24.67	53.87	**8.11**
Tiger	73.86	47.89	68.69	82.18	174.02	101.43	*13.00*	**11.92**
Walking	79.6	292.16	211.02	16.12	*3.38*	**2.62**	6.33	4.76
Walkman	71.84	34.88	33.86	16.15	**6.45**	*7.30*	7.68	8.39
Average	54.39	61.53	53.13	23.56	33.72	*21.67*	40.93	**7.78**

**Table 4:** Average overlap rate. The best two results are shown in **Bold** and *Italics* fonts.

Algorithms	KMS [10]	EMshift [12]	SOAMST [19]	CBWH [20]	ASLA [51]	SCM [52]	CT [53]	Our
Basketball	0.25	0.33	0.42	**0.74**	0.06	0.23	0.2	*0.66*
Board	0.5	0.29	0.32	0.65	**0.76**	0.64	0.37	*0.72*
Boy	0.29	0.1	0.28	*0.74*	**0.77**	0.37	0.65	0.73
Car	0.21	0.14	0.09	*0.75*	**0.77**	0.72	0.59	0.74
Caviar	0.72	0.12	0.08	0.57	0.44	**0.91**	0.21	*0.77*
Couple	0.06	0.08	0.36	0.43	*0.65*	0.09	0.57	**0.68**
Crossing	0.09	0.39	0.18	0.67	**0.79**	*0.76*	0.70	0.70
Deer	0.13	0.1	0.22	*0.66*	0.64	0.59	0.04	**0.71**
Football	0.25	0.25	0.26	0.56	0.60	**0.79**	0.42	*0.71*
Doll	0.25	0.15	0.26	*0.61*	0.42	0.50	0.15	**0.68**
Girl	0.28	0.29	0.34	0.35	0.12	**0.69**	0.26	*0.64*
Jumping	0.08	0.03	0.02	0.07	*0.72*	**0.73**	0.05	0.68
MountainBike	0.44	0.08	0.03	*0.65*	0.41	**0.68**	0.14	0.64
Occlusion	0.23	0.47	0.38	0.65	*0.80*	**0.86**	0.42	0.62
Singer	0.07	0.1	0.16	0.34	**0.74**	*0.71*	0.36	0.58
Subway	0.11	0.13	0.2	0.55	**0.71**	*0.70*	0.58	0.69
Sylvester	0.26	0.11	0.06	0.52	*0.63*	0.52	0.29	**0.68**
Tiger	0.16	0.12	0.19	0.14	0.17	0.09	*0.59*	**0.66**
Walking	0.19	0.07	0.06	0.48	**0.71**	*0.69*	0.56	*0.69*
Walkman	0.08	0.2	0.18	0.53	*0.67*	**0.68**	0.61	0.64
Average	0.23	0.18	0.2	0.53	0.58	*0.60*	0.39	**0.68**

### Qualitative Evaluation

All the sequences involve at least one critical challenge. Thus, we divide all the sequences into six groups according to their main challenge and qualitatively evaluate the tracking results of these sequences in six different ways as follows.

Experiments on the data with partial occlusion: Occlusion is one of the most general crucial problems in tracking issue. We test the algorithms on 3 sequences with target occlusion. From the results of the Occlusion sequence shown in Fig. 5, the KMS algorithm becomes worse when the occlusion area gets bigger, and the size of bounding box decreases with the increasing of the occlusion area. This can be explained that the color of the skin takes a large part in the color based histogram in this sequence. Meanwhile, the scale strategy in KMS is to choose the best one in 3 times calculations of 10% scale changing. This is the reason why the bounding box decreasing. The CBWH algorithm performs quite well when occlusion area is not bigger than half of the face. But it begins to lose the target when more than half of the face is occluded. In the Caviar sequence, the target is occluded by two people one after another. The SOAMST and the ASLA algorithms are influenced by the man who is parallel to the target. Meanwhile, the CBWH is affected by the reflections on the floor and the bounding box drifts upward obviously. Furthermore, the KMS, SCM and our algorithm can track the target well after the occlusion. Nevertheless our method can get a better result in quantitative value. In the Subway sequence, the target is a man who wears dark clothes and walks from left to right of the scene. During the process, the man is occluded by several people. It can be observed in Fig. 5 that all the mean shift based algorithms lost the target in the procedure except ours. The proposed method can accurately locate the targets as our target location is computed by the corresponding feature points by image matching. In addition, the SCM and ASLA methods also achieve good performance in most cases since both of them contain part based representations with overlapping patches.

Experiments on the data with illumination variations: We test several sequences (Singer, Sylvester, Tiger) with dramatic illumination change. In the Singer sequence, the light of the scene changes dramatically and the camera is pulling away from the stage. Most of the mean shift based algorithms fail to track when the light changes. In contrast, our method achieves satisfactory performance in this sequence. However, it also appeared some jitter because it cannot detect enough feature points in the process of illumination change. In the Sylvester sequence, the target is a toy cat being waved through the light intermittently. Moreover, the target is rotated by the man at times. As shown in Fig. 5, the EMshift and SOAMST are failed in the beginning and the bounding box becomes larger and larger as the tracking goes. The CBWH algorithm can handle with this situation until frame 1100 and the target is lost in a dark place. Both the ASLA algorithm and ours can successfully track the target over the whole sequence. However, our method shows outstanding performance obviously both in center error and overlap rate. This can be explained that distinguishing descriptor in our feature extraction strategy and the matching ability of DAISY descriptor in light change are quite robust. The same problem is more obvious in the Tiger sequence which is similar to the Sylvester sequence. The target is a toy tiger which is waved around the light and hidden behind the plant at times. Besides, occlusion and abrupt motion both occur in this sequence, all the other algorithms are not able to track the target well. In contrast, the CT and the proposed algorithm track the target successfully for the entire sequence. Moreover, our tracker outperforms the other methods in all the metrics. The principal reason is that we use the searching extending strategy to cope with the fast moving target.

Experiments on the data with in-plane or out-of-plane rotation: The target in Mountain-bike sequence is a man riding a bike from right to left. In the whole process, the man jumps in the air, then rotate his body and land in the end. Only the CBWH, SCM and the proposed algorithm can be successful in tracking the target in the whole sequence. In the Couple sequence, the target moves fast and rotates in the process. The KMS and EMShift methods lost the target in the beginning. Notice that the other algorithms drift to the car near the target (e.g., frame 91) except the SCM and our method. Then, when the target near the car, the SCM algorithm does not catch the target neither. The proposed method performs well for the target with non-rigid pose variation and out-of-plane rotation in this sequence. It can be attributed to the fact that the histogram of our tracker is based on local features which are robust. The Girl sequence consists of both in-plane and out-of-plane rotations. Only the SCM and the proposed method success in tracking the target and the other algorithms fail when the girl rotates her head. Compared with the SCM algorithm, our tracker drifts upward when the girl back to us. Because there is no histogram update scheme in our method, only the feature points of the head silhouette can be utilized in that case. This will be a focus of future work.

Experiments on the data with scale variations: In the Walking sequence, the KMS, EMShift and SOAMST methods fail to track the target due to their scale strategies. The other algorithms can handle with this sequence. The Walkman sequence shows a man walks in the scene with scale variation and out-of-plane rotation. It can be seen from the samples in Fig. 5 that our tracking results are accurately located on the true target without drift. In addition, the CBWH and the non-mean shift based algorithms can also track the target well. The Doll sequence is quite long and has 3872 frames. Similar to the Sylvester sequence, the target is held by a man and waved with occlusion, rotation and scale variation. The CBWH, SCM and our method achieve stable performance in the entire sequence. Thus, it is proved that our method is robust to deal with the long time tracking as well as the state-of-the-art algorithms.

Experiments on the data with motion blur and fast moving: Generally, the targets with motion blur and fast moving are hard to catch in object tracking. The first testing data is the Deer sequence, where the target is a jumping deer’s head among a lot of deer. Most tracking algorithms are not able to follow the target at the beginning of this sequence because the target object is almost indistinguishable and moves fast. The CBWH, ASLA, SCM and our algorithm successfully track the target object throughout the entire sequence. However, as shown in Fig. 5, frame 30 and 56 show that the CBWH method mistakenly locates a similar deer head instead of the correct one. The reason is that the CBWH method based on color histogram cannot distinguish the target from the similar background. In addition, the proposed algorithm achieves low tracking error and high overlap rate due to that its histogram is created by distinguishing feature points. In the Boy, Football and Jumping sequences, it is difficult to predict the locations of the tracked objects when they undergo abrupt and fast motion. We can see that the CBWH method successes in tracking the target in both the Boy and the Football sequences. But it fails in the Jumping sequence. Meanwhile, for the non-mean shift based methods, their local features and update strategy can help them to deal with this situation. However, only the proposed method successfully keeps track of the targets in all the sequences. It can be attributed to our DAISY feature which can get good matching result in blurred images and the searching extending strategy which can deal with the fast moving object.

Experiments on the data with background clutters: The Board sequence is challenging because the target is blurred with rotation and the background is cluttered. Our method performs well in this sequence as the target can be differentiated from the cluttered background due to our feature points based histogram. In addition, the scale strategy is successful in estimating the actual scale of the target. The Basketball sequence is difficult to track because there are 4 more players wearing the same jerseys as the target. Meanwhile, the target is occluded by the other players or the referee and the flashlight also occurs in the scene (e.g., frame 678). It can be seen from frame 484, 561 and 678, that all the other algorithms drift to another player when the target undergoes the occlusion or illumination variation. In contrast, the proposed method is able to track the right target accurately in the entire sequence due to the discriminative features and the mean shift iteration process. The Car and the Crossing sequences are similar, and the sequences are parts of traffic surveillance videos. In both sequences, the targets are inconspicuous as they are indistinguishable from the background. These experiments also test the ability of algorithms for tracking the low contrast targets. For the non-mean shift based methods, they all perform quite well in these sequences. For the mean shift based algorithms, although the motion trails of the targets are very simple, the KMS algorithm cannot distinguish the target from the similar background and fails to track. The same problem happens to the EMShift and the SOAMST algorithms. The CBWH method can track the target in both sequences because it uses the corrected background weighting histogram to make its histogram more distinguished. Meanwhile, the proposed method works stably and has a good performance in this situation.

### Running-time Evaluation

We also measure the speed of the algorithms using the average processing frames per second. Note that the algorithms are not implemented in the same language(some using MATLAB with MEX files, some using pure MATLAB) which may bias the speed measurement towards the more efficient programming language. Here we compare all the algorithms mentioned above and show the results in Table 5. The results are calculated from all the 20 sequences on a 1.6GHz Intel(R) Pentium(R) PC. It can be seen from Table 5 that our algorithm cannot archive the real-time request (24 FPS), but it runs faster than several state-of-the-art methods using the same coding environment.

**Table 5:** Average running time. (Code: M—Matlab; MC—Matlab with C/C++ MEX files)

Algorithms	KMS [10]	EMshift [12]	SOAMST [19]	CBWH [20]	ASLSA [51]	SCM [52]	CT [53]	Our
Code	M	M	M	M	MC	MC	MC	M
FPS	5.18	9.65	7.40	113.37	2.50	0.42	141.24	9.80

## Conclusion

In this paper, we propose a novel mean shift based algorithm in video target tracking. First, we use the FAST detector and DAISY descriptor to extract the image features, then utilize them to create the target model histogram and the target candidate histogram. Both the histograms are then computed iteratively in the mean shift process to locate the object. Experimental results compared with several state-of-the-art methods on challenging sequences demonstrate the effectiveness and robustness of the proposed algorithm. In addition, our method can deal with the target appearance change which is not drastic. However, it is difficult to keep on tracking without the histogram update scheme when the target totally changed. Thus, the focus of our future work is introducing the histogram update strategy. Meanwhile, solving the full occlusion problem is also our plan for the next step.
